# Eddy Current Pulsed Thermography with Different Excitation Configurations for Metallic Material and Defect Characterization

**DOI:** 10.3390/s16060843

**Published:** 2016-06-08

**Authors:** Gui Yun Tian, Yunlai Gao, Kongjing Li, Yizhe Wang, Bin Gao, Yunze He

**Affiliations:** 1 School of Automation Engineering, University of Electronic Science and Technology of China, Chengdu 611731, China; g.y.tian@uestc.edu.cn (G.Y.T.); wangyizhe617525@163.com (Y.W.); bin_gao@uestc.edu.cn (B.G.); 2 School of Electrical and Electronic Engineering, Newcastle University, Newcastle upon Tyne, NE1 7RU, UK; k.li3@newcastle.ac.uk (K.L.); hijacker@163.com (Y.H.); 3 College of Automation Engineering, Nanjing University of Aeronautics and Astronautics, Nanjing 211106, China; 4 College of Electrical and Information Engineering, Hunan University, Changsha 410082, China

**Keywords:** eddy current pulsed thermography (ECPT), material characterization, nondestructive evaluation (NDE), multi-physical phenomenon, excitation configuration

## Abstract

This paper reviews recent developments of eddy current pulsed thermography (ECPT) for material characterization and nondestructive evaluation (NDE). Due to the fact that line-coil-based ECPT, with the limitation of non-uniform heating and a restricted view, is not suitable for complex geometry structures evaluation, Helmholtz coils and ferrite-yoke-based excitation configurations of ECPT are proposed and compared. Simulations and experiments of new ECPT configurations considering the multi-physical-phenomenon of hysteresis losses, stray losses, and eddy current heating in conjunction with uniform induction magnetic field have been conducted and implemented for ferromagnetic and non-ferromagnetic materials. These configurations of ECPT for metallic material and defect characterization are discussed and compared with conventional line-coil configuration. The results indicate that the proposed ECPT excitation configurations can be applied for different shapes of samples such as turbine blade edges and rail tracks.

## 1. Introduction

Eddy current pulsed thermography (ECPT) [1], as an emerging nondestructive evaluation (NDE) method, has been investigated for detection and characterization of structural degradation. Material degradations and failures such as defect, fatigue, corrosion, and residual stress have been detected and evaluated using ECPT [2,3,4,5,6,7]. The configuration of ECPT is one of the major issues for induction thermography, which greatly respond to the heating effect and evaluation of structural states from thermal image sequences. Wilson *et al.* [2] proposed a line-coil-based excitation to inductive heating on sample surfaces for rolling contact fatigue crack detection. Tian *et al.* [5] employed a rectangular single turn coil to evaluate the contact fatigue of gear. Cheng *et al.* [3] investigated rectangular multi-turns coil of ECPT to detect surface cracks of carbon-fiber-reinforced plastic (CFRP) materials. There are some limitations associated with these excitation configurations. The first limitation is the influence of non-uniform heating, which means the abnormal defect is covered by a severe temperature gradient. One solution is scanning thermography [8], which makes uniform heating by a constant moving of coil. However, the scanning configuration makes the system complicated. Another solution is the application of phase information through eddy current pulsed phase thermography (ECPPT) based on an FFT algorithm [9,10] and thermal pattern separation and analysis [6,11]. However, these algorithm innovations cannot eliminate the non-uniform heating from the sources. The second limitation is the field of view, which is blocked by the coil. A possible solution is the configuration of the transmission mode where the infrared (IR) camera and coil are placed on opposite sides [12]. However, the transmission mode is not suitable for most metallic components due to the great thickness. An alternative solution is the use of lateral heat conduction excited by line coil [13]. This approach, however, has a directional sensitivity and is only sensitive to the cracks which are parallel to the coil. In order to solve these problems, Li *et al.* [4] proposed the Helmholtz coil excitation of ECPT for the state detection of bond wires in an insulated gate bipolar transistor (IGBT) sample. Peng *et al.* [14] investigated the Helmholtz-coil-type excitation of ECPT to produce approximate uniform heating on railhead surfaces and provide a larger detection area without the coil shielding problem. The Helmholtz coil excitation configuration does achieve the uniform heating and open-view imaging. After analyzing the principle of electromagnetic excitation, it is speculated that the ferrite-yoke-based excitation configuration is an effective means for uniform heating and open-view imaging. This idea derives from magnetic flux leakage (MFL) [15], where Gao *et al.* proposed a ferrite-yoke-based model to combine MFL and ECPT after a comparison of multiple crack evaluations.

With the development of ECPT [15,16,17] for challenging nondestructive evaluation (NDE) applications such as free-form samples, multiple cracks such as Rolling Contact Fatigue Cracks (RCFs) and Stressed Corrosion Cracks (SCCs), gears and bearings, the limitations of non-uniform heating, and restricted views have been the some of the greatest obstacles in the application of ECPT. To overcome a non-uniform induction field due to sample geometry, e.g., defects at edges or corners and the block view of excitation coils, this paper introduces two recently developed ECPT configurations: Helmholtz-coil-based ECPT for the uniform heating of free-form samples and ferrite-yoke-based ECPT with magnetic flux and eddy current thermography for ferromagnetic material uniform heating and open-view infrared imaging. After a comparison of these configured ECPTs through simulation and experimental results, the multi-physics and characteristics of them are discussed for inverse problems and the quantitative NDE of metallic materials. The rest of this paper is organized as follows: Section 2 introduces theory background and methodology for two different excitation configurations. Section 3 describes the numerical simulations and experimental studies results of different ECPT configurations, the multi-physical effect of hysteresis losses, stray losses, and eddy current heating are conducted and implemented for ferromagnetic and non-ferromagnetic materials. After a comparison and discussion of different ECPT configurations, a conclusion and future works are derived in Section 4.

## 2. Theory and Methods

### 2.1. Theory Background of ECPT

**Figure 1:**
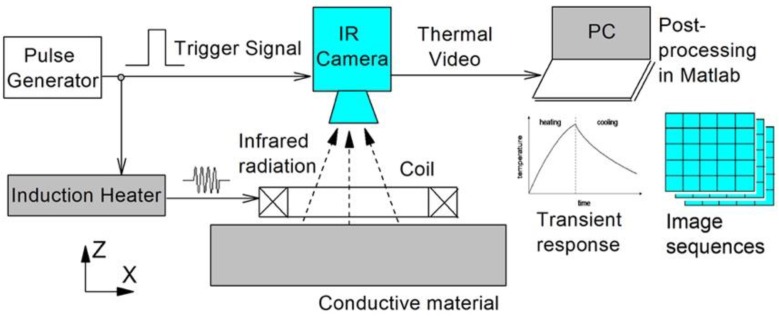
Schematic diagram of eddy current pulsed thermography (ECPT).

ECPT involves multi-physical interactions [17] with electromagnetic-thermal phenomena including induced eddy currents, Joule heating, and heat conduction. Figure 1 shows a schematic of the operation principle of ECPT for inspecting metallic components. After being triggered by a pulse signal from a pulse generator, the induction heater generates an excitation signal, which is a period of high-frequency alternating current with high amplitude. The current is then driven into an inductive coil positioned on a sample with a small distance. When the current passes through the coil, it induces eddy currents in the components. These eddy currents are governed by a subsurface penetration depth, *δ*, based on an exponentially damped skin effect. The latter can be calculated from

(1) $$\delta ={\left(\pi \mu \sigma f\right)}^{-1/2}$$

where *f* is the frequency of the excitation signal, *σ* is the sample electrical conductivity (S/m, siemens per meter), and *μ* is the magnetic permeability (H/m, henries per meter) of the sample. The temperature of conductive material increases due to resistive heating from the induced eddy current, which is known as Joule heating. It can be expressed by the equation:

(2) $$Q=\frac{1}{\sigma }{\left|J_{s}\right|}^{2}=\frac{1}{\sigma }{\left|\sigma E\right|}^{2}$$

where the sum of generated heat Q is proportional to the square of the eddy current density, $J_{s}$. It is concluded that skin depths for metallic materials is very small (about dozens of μm under 100-kHz excitation). Thus, the heating style for metallic materials is surface heating [8]. The resistive heat will diffuse as a time transient until a heat balance is restored between the bulk and its surface, at which time the signal will come to a steady state. The heat diffusion process is governed by

(3) $$\frac{\partial T}{\partial t}=\frac{\lambda }{\rho C_{p}}\left(\frac{\partial^{2}T}{\partial x^{2}}+\frac{\partial^{2}T}{\partial y^{2}}+\frac{\partial^{2}T}{\partial z^{2}}\right)+\frac{1}{\rho C_{p}}q\left(x,y,z,t\right)$$

where T=T(x,y,z,t) is the temperature distribution, λ is the thermal conductivity of the material, ρ is the density, and $C_{p}$ is the specific heat, and q(x,y,z,t) is the internal heat generation function per unit volume and unit time. In this process, the temperature field on the surface of components will be captured by an IR camera as a sequence of an image saved on a PC. One typical temperature response at a pixel is shown in Figure 1. It can be divided into two phases: the heating phase and the cooling phase. If there is a defect, these physical processes will be interrupted. The aforementioned is the basic principle of ECPT, which is also illustrated in Figure 1.

When an induced eddy current encounters geometric discontinuity or local anomalies, the eddy current will be forced to divert, resulting in distorted regions of eddy current densities. The eddy current diversion leads to heat distribution with different thermal contrasts between defective and defect-free area, which makes the defect visible using an IR camera. The thermal transient responses during the heating and cooling stages include rich pattern information, which can be employed to evaluate crack detection and classification, and structural degradation through thermal image sequences. However, current ECPT with single line-coil excitation [1,2,3] is not good enough for complex geometry structures, e.g., gears, turbine blades, rail tracks, *etc.* Different configurations of ECPT are introduced and compared under different test samples.

### 2.2. The Methodology of the Proposed Configurations

**Figure 2:**
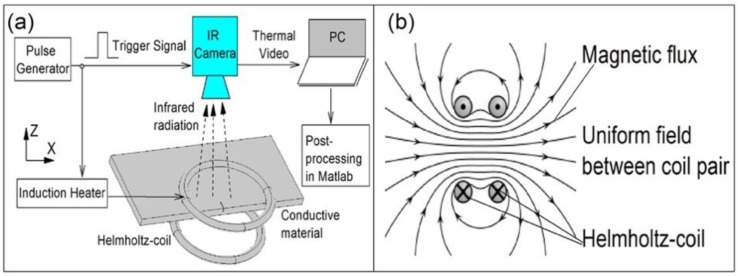
Proposed Helmholtz-coil configuration of ECPT. (**a**) Schematic diagram of Helmholtz-coil-based ECPT system. (**b**) Magnetic flux distribution with uniform field between two coils.

This paper proposes two different ECPT configurations with Helmholtz-coil- and ferrite-yoke-based structures to improve the capability of ECPT through uniform field excitation for inductive heating and open-view imaging. The schematic diagram of the Helmholtz-coil configuration of ECPT with magnetic flux distribution is illustrated in Figure 2. The Helmholtz coil is known to generate a uniform magnetic field in a wide region around a center point of the coil pair axis, as shown in Figure 2b. Compared with line-coil-based ECPT, here, the Helmholtz-coil is employed to generate super heating patterns in the sample or sample edges between two coils for defect evaluation. The defects in the sample or sample edges are detected through a thermal contrast from the original uniform field or heat distribution, which can be recorded by an IR camera towards the open-view space between two coils.

**Figure 3:**
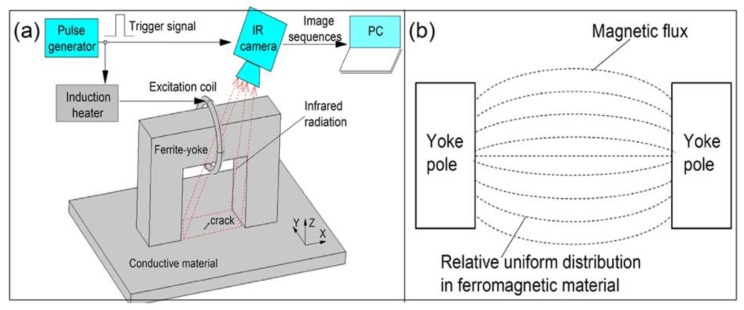
Proposed ferrite-yoke-based configuration of ECPT. (**a**) Schematic diagram of ferrite-yoke-based ECPT system. (**b**) Magnetic flux distribution in ferromagnetic material between yoke poles.

The schematic diagram of the ferrite-yoke-based configuration of ECPT and its magnetic flux distribution are illustrated in Figure 3. This configuration combines both the advantages of magnetic flux leakage and ECPT. The magnetic flux generated by the circle excitation coil is transmitted to the sample surface and near the surface between the two poles of yoke through a magnetic circuit, including ferrite-yoke, the air gap, and the sample. The magnetic flux is relatively uniformly distributed in the ferromagnetic material, which leads to a relatively uniform eddy current field orthogonal to the magnetic flux, according to the Faraday's law for electromagnetic induction. Meanwhile, both the magnetic flux and eddy current generate heating through hysteresis loss and eddy current loss in ferromagnetic samples due to alternating dynamic magnetization. The leakage magnetic field induced by the interactions of the applied field and cracks also lead to stray losses for different heating responses around defects. Thermal contrast between defective and defect-free areas can be imaged with an IR camera in open-view, which avoids line-coil blocks in the current ECPT.

## 3. Simulation and Experimental Studies

As described in Section 2, three ECPT excitation configurations are illustrated. To understand their operational mechanism, behind the multi-physical coupling effect and characteristics for NDE, simulations and experiments were conducted with different samples. A typical ECPT system with a line-coil- and ferrite-yoke-based ECPT were applied for ferromagnetic material. Helmholtz-coil-based ECPT was applied to a non-ferromagnetic sample. The different ECPT structures and their advantages and disadvantages were investigated and identified.

### 3.1. Simulation and Results

**Figure 4:**
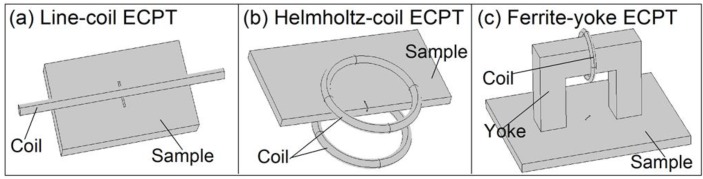
Numerical modeling for ECPT simulation with different configurations. (**a**) Line-coil ECPT. (**b**) Helmholtz-coil ECPT. (**c**) Ferrite-yoke-based ECPT.

Numerical modeling for ECPT simulations with different configurations was established in COMSOL Multiphysics 4.3b. Three different configurations of ECPT are illustrated in Figure 4. The geometrical and material physical parameters, e.g., permeability, electrical and thermal conductivity of the sample, and excitation are referenced in [4,14,15]. For comparison, three types of coil configurations and defects in different samples as illustrated in Figure 4 were simulated, e.g., a surface crack in steel_1008 under a line-coil with 0.5 mm of lift-off, as shown in Figure 4a, a crack edge in Ti-alloy between two coils, as shown in Figure 4b, and an angular crack at 45° in steel_1008 between two poles of ferrite-yoke, as shown in Figure 4c. The size of the sample is 150 mm in length, 90 mm in width, and 10 mm in depth. The size of the crack is 10 mm in length, 5 mm in depth, and 0.5 mm in width. The inner diameter of the Helmholtz-coil and circular excitation coil wound on the yoke is 50 mm with a wire outer diameter of 6.35 mm. The refined mesh is setup for the calculation of a 3D model. The current 380 A/256k Hz flows into the excitation coil to generate magnetic flux for induction heating. The duration of the heating is 200 ms, which is enough to generate an effective thermal response for defect evaluation.

**Figure 5:**
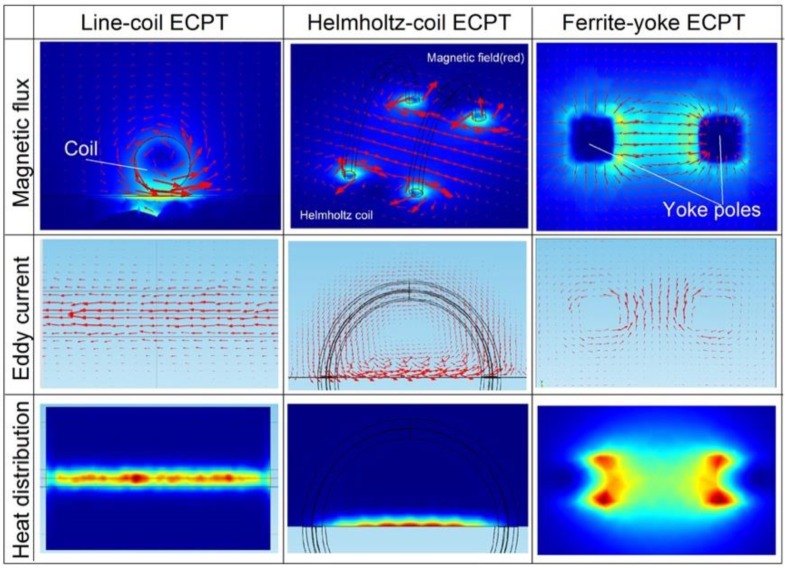
Simulation results of ECPT with different configurations such as line-coil-, Helmholtz-coil-, and Ferrite-yoke-based excitation on a multi-physical-phenomenon and coupling effect.

After a short period of 100 ms for induction heating, simulation results, which can typically demonstrate the operational characteristics of the three proposed ECPT configurations, were obtained and are illustrated in Figure 5. The multi-physical interactions and coupling effect were analyzed and are discussed with the magnetic flux, the induced eddy current, and the heat distribution for the defect evaluation. In a typical ECPT system in the first column of Figure 5, line-coil ECPT generates a magnetic flux (red vectors) around the coil and interacts with the sample with a limited area close to the coil, as shown in the side view of the top-left image. The induced eddy current is parallel to the coil direction and distributed in the sample close to the line-coil; hence, it generates a limited heating area along with the line-coil. In the middle column of Figure 5, the Helmholtz-coil generates a uniform magnetic field (red vectors) between two coils, as shown in the top-middle image. The eddy current is orthogonal to the magnetic flux and parallel to the sample edge with a relatively uniform distribution, which is positioned in the center of the coil pair. This leads to a relatively uniform heating area around the sample edge, as shown in the bottom-middle image. In the right column of Figure 5, a ferrite-yoke-based configuration generates a relatively uniform magnetic field between two poles of yoke with the orthogonally distributed induction eddy current. The heating response of this configuration distributed on a large scale between yoke poles accompany four heater areas around the yoke corner, as shown in the bottom-right image in Figure 5.

**Figure 6:**
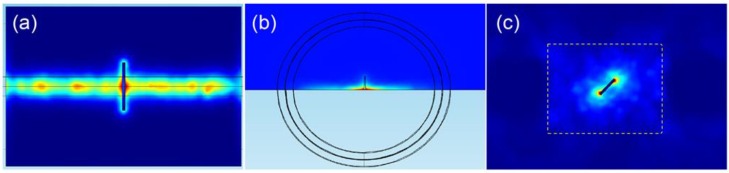
Simulation results of ECPT with different configurations. (**a**) Line-coil ECPT for the crack, (**b**) Helmholtz-coil-based ECPT for the crack edge. (**c**) Ferrite-yoke-based ECPT for the angular crack.

To verify the capabilities of the three different ECPT configurations for the defect evaluation, different defects were employed, and the simulation results are illustrated in Figure 6. As shown in Figure 6a, the crack profile is demonstrated through thermal contrast, and heat density around the crack is decreasing away from the excitation coil. The strong thermal responses under the line-coils are partially blocked in practical applications, so it is difficult to have a full picture of crack responses. As shown in Figure 6b, the crack edge is clearly identified by the heat concentration in the crack open position and the heat distribution around the crack edge. Even heat density is decreasing away from the sample edge. As shown in Figure 6c, the angular crack is presented through the heat distribution around the crack tips with the heater area and the thermal contrast around the crack edges.

### 3.2. Experiments and Results

**Figure 7:**
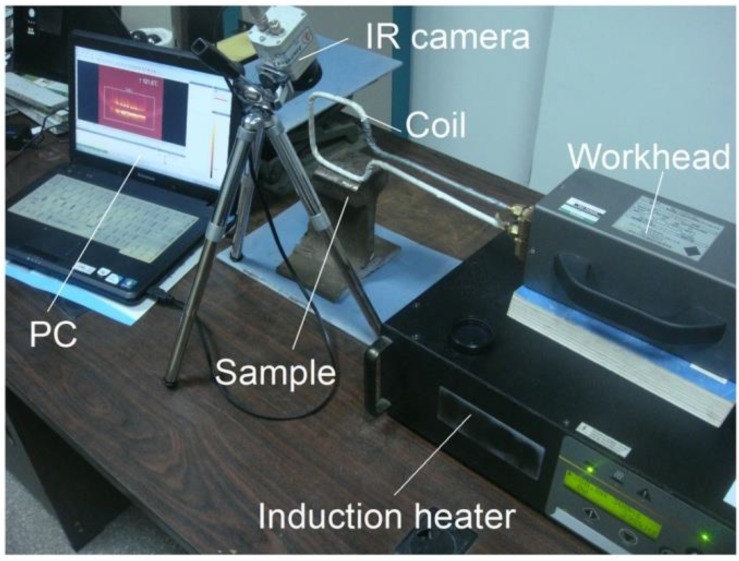
Experimental system of ECPT.

**Figure 8:**
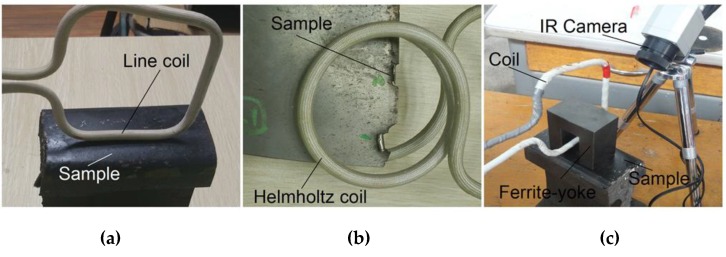
ECPT with different configurations. (**a**) Line-coil ECPT. (**b**) Helmholtz-coil ECPT and (**c**) Ferrite-yoke-based structure for ECPT.

To verify the simulation above and characteristics of the three configurations of ECPT for defect identification, experiments were conducted with the ECPT system including the Easyheat224 induction heater, the high conductive hollow copper tube coil, a water-cooling device, the Optris PI400 IR camera (Optris GmbH, Berlin, Germany), and a PC, the detailed parameters of which are referenced in [15]. Three types of configurations with the line-coil-, Helmholtz-coil- and ferrite-yoke-based structures were employed, the coil parameters of which were the same as those of the simulation. Two samples with rail specimens of ferromagnetic material, including multiple cracks and blade specimens of Ti-alloy material including natural edge defects, were used for the test. As shown in Figure 7, the ECPT system structures, parameters, and test procedures were the same as [15]. The excitation current 380 A/256k Hz was used for the induction heating. The temperature resolution of the Optris PI400 IR camera was 0.08 K with a sensing spectral range of 7.5–13 μm. The size for the focus spot about 150 mm×120 mm area was used for the thermal imaging of the defect. A frame rate of 80 Hz for the IR camera with a 382×288 array was used to record thermal images within a 200-ms duration of heating. In Figure 8 three different ECPT excitation configurations are illustrated with different metallic samples and defects, e.g., one edge defect in one blade from the Nanjing University of Aeronautics and Astronautics (NUAA) and the RCF cracks in the rail, which is the same sample used in [2,14,15]. The RCF multiple cracks are distributed in a gauge corner of the rail head with complex surface shapes, where the crack lengths are up to 20 mm, the distances are about 0.5–2 mm among multiple RCF cracks, and the orientation angles are about 45°–60° against the rail travel direction. The edge defects in the blade include two distinct natural notches with lengths of about 5–8 mm along the blade edge and depths of about 1–5 mm inside the blade.

**Figure 9:**
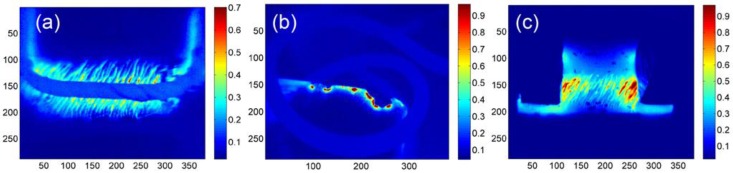
Experimental results of ECPT with different configurations. (**a**) Line-coil for multiple cracks. (**b**) Helmholtz-coil for crack edges and (**c**) Ferrite-yoke excitation for multiple cracks.

During experiments, thermal images were obtained by using the IR camera for crack identification. The results after a heating duration of 100 ms were selected for analysis and discussion, as shown in Figure 9, which is identical to all simulation results. As shown in Figure 9a, rail multiple cracks are clearly demonstrated through thermal contrast between defective and defect-free areas, but defects under line-coils could not be imaged due to the coil blocking effect. The heating density was also decreasing away from the line-coil. As shown in Figure 9b, the natural edge defect profile in the blade sample is well presented by IR imaging towards the sample through open-view imaging between the two coils. The defect-free heating area is relatively uniformly distributed. As shown in Figure 9c, rail multiple cracks are fully demonstrated in larger areas between yoke poles with open-view imaging under relatively uniform heating. Compared with the three images in Figure 9, the heating uniformity and open-view imaging area of Figure 9c are better than Figure 9a,b. Two new configurations of ECPT with Helmholtz-coil- and ferrite-yoke-based excitation demonstrate better characteristics for complex structural edge defect detection and RCF multiple crack visualization without a blocking effect than conventional line-coil ECPT. Comparing Figure 9c with the previous results with ECPT based on lateral heat conduction [13], the advantages of the open view are apparent. Three tests can view multiple cracks and defects at edges. As illustrated in Figure 9c, the heating distribution pattern near the ferrite-yoke poles has stronger heating than areas, e.g., in the central area, as simulated in the bottom of the right column of Figure 5, which will be further evaluated for quantitative NDE.

**Figure 10:**
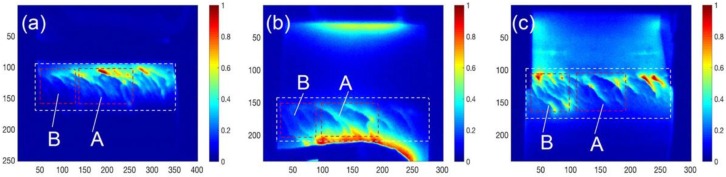
Experimental results of ECPT for the same sample with RCF multiple cracks using (**a**) line-coil. (**b**) Helmholtz-coil and (**c**) ferrite-yoke excitation configurations.

For quantitative comparison of three different ECPT excitation configurations, experimental results on the same sample with RCF multiple cracks are demonstrated in Figure 10. The signal-to-noise ratio (SNR) [18,19] or thermal pattern contrast are calculated in different defective regions to evaluate performance of defect detection and characterization of three ECPT configurations with each other. SNR describes the thermal contrast between defective region considered as “signal” and non-defective region defined as “noise”. The calculation of SNR in dB is defined below:

(4) $$SNR=\frac{S}{N}=20log_{10}\left(\frac{T_{mD}-T_{mN}}{\sigma \left(T_{mN}\right)}\right)\;\left[dB\right]$$

where $T_{mD}$ and $T_{mN}$ are average temperature in defective and non-defective regions, respectively. The $\sigma \left(T_{mN}\right)$ is the temperature standard deviation in the relevant non-defective regions. The defective regions are marked in Figure 10 including the defective region with dotted line enclosed and two local defective regions A and B. Relevant non-defective regions selected are same areas for the three configurations. The SNR values of these selected regions are illustrated in Table 1 for quantitative comparison of three ECPT configurations.

As shown in Figure 10a, rail multiple cracks can be identified by thermal contrast in the regions close to line-coil. However, thermal patterns are influenced by the line-coil non-uniform heating, which are shown that higher thermal intensity close to coil and lower intensity with the distance away from coil. It also can be proved by the results that SNR values of the region A are better than the region B, which is away from coil, as illustrated in Table 1. As shown in Figure 10b, a relatively uniform heating area is produced between Helmholtz-coil pairs for defect detection. It proves better performance for blade edge defects inspection as shown in Figure 6b and Figure 9b. However, the defective region close to coil is also influenced by the non-uniform heating that higher thermal intensity close to coil as shown in Figure 10b. The similar SNR values to line-coil also prove its non-uniform influence on defect detection and characterization. Because of the uniform heating and open-view imaging of ferrite-yoke excitation of ECPT, the whole defective regions are clearly demonstrated with better thermal contrast as shown in Figure 10c. The higher SNR values of ferrite-yoke excitation also prove the better defect characterization than other two because of its superior characteristics. Overall, it is identified that different ECPT configurations own individual strengths for different shape samples and defects characterization. Recent improvement of ECPT with multi-physical fields interactions for uniform heating and open-view imaging provide potential capabilities for quantitative evaluation of complex metallic structures.

Based on the above simulation and experimental results, all the crack orientations can be detected by these three ECPT configurations [13,14,15,16]. The perpendicular and oblique cracks produce large electrical resistance to change the distribution of induced eddy currents, which are flowing parallel to line-coil and blade edge as shown in Figure 5. The parallel cracks can be identified through lateral heat conduction as described in [13]. Thermal patterns for these defects have been illustrated in Figure 6, Figure 9 and Figure 10 to prove defect orientations characterization. The limitations of line-coil excitation are the blocking effect and non-uniform heating that influence the defect detection and characterization. The Helmholtz-coil and ferrite-yoke have been proved suitable for complex shaped edge and multiple cracks inspection as shown in Figure 9 and Figure 10, respectively, with higher SNR values. However, the defect detection and characterization of Helmholtz-coil is still influenced by its non-uniform heating in the region close to the Helmholtz-coil. The ferrite-yoke excitation is the best one in these three ECPT configurations because of the superior characteristics and high SNR values for the two regions. Similarly, its non-uniform heating regions also exist in the area close to the ferrite-yoke poles. The different configurations of ECPT can be applied for metallic material and defect characterization e.g., material stress [20,21], different defects e.g., fatigue [5], RCF [14,15], corrosion [22] and stress corrosion crack (SCC) via multiple physics characterization and fusion [17,23]. Relative uniform excitation through Helmholtz-coil or ferrite-yoke-based excitation provides better thermal images for the material characterization including quantitative NDE.

## 4. Conclusions and Future Works

This paper reports three different excitation configurations of ECPT systems for complementary NDE of complex metallic structures and defects characterization. Simulation and experimental results demonstrate different ECPT configurations own individual strengths for different shape samples and defects detection applications. One proposed configurations of ECPT with Helmholtz coil with relatively uniform inductive heating can be applied for crack edge [4,24] detection, and other proposed ferrite-yoke-based excitation can be applied for the characterization of RCF cracks in the rail track heads [14,15] with uniform heating and open-view thermal imaging. The open-view field imaging can be applied for large area inspection in contrast to the ECPT with the line-coil. A quantitative comparison of three ECPT configurations through SNR values is also provided to evaluate the defect detection and characterization performance.

In conclusion, the relatively uniform area of ECPT configuration with the Helmholtz coil can easily be affected by the inspected sample inside the coil. Reasonably small samples can be inspected. For the ECPT configuration using ferrite-yoke for excitation can robustly control the uniform excitation field and direction. The inspected samples affecting the excitation field is relative small. The linear coil excitation can reach a local maximum with a strong influence on the surface curvature of the tested samples.

Although rail multiple cracks and blade natural edge defects have well detected and verified the capabilities of differently configured ECPTs, quantitative NDE needs further consideration and processing due to the geometric superimposition on the heating pattern [23,25,26]. Their inspection limitations of electro-conductive materials only, constant lift-off, and the requirement of sample scanning need to be further addressed.

## Figures and Tables

**Table 1:** Defect detection performance comparison of different ECPT excitations with SNR in dB.

	Region	SNR (dB) of Whole Defective Area	SNR (dB) of Region A	SNR (dB) of Region B
Excitation				
Line-coil		4.88	8.42	2.42
Helmholtz-coil		3.86	8.33	4.03
Ferrite-yoke		10.53	7.45	13.31
